# High Aspect Ratio Nanoimprint Mold-Cavity Filling and Stress Simulation Based on Finite-Element Analysis

**DOI:** 10.3390/mi8080243

**Published:** 2017-08-06

**Authors:** Hongwen Sun, Minqi Yin, Haibin Wang

**Affiliations:** 1 College of Internet of Things Engineering, Hohai University, Changzhou 213022, China; hhucymq@163.com (M.Y.); 20021646@hhu.edu.cn (H.W.); 2 Jiangsu Key Laboratory of Power Transmission and Distribution Equipment Technology, 200 JinLing Road North, Changzhou 213022, China

**Keywords:** nanoimprint lithography, high aspect ratio, mold, cavity filling, stress, finite-element analysis

## Abstract

High aspect ratio three-dimensional micro- and nanopatterns have important applications in diverse fields. However, fabricating these structures by a nanoimprinting method invites problems like collapse, dislocation, and defects. Finite-element analysis (FEA) is a good approach to help understand the filling process and stress distribution. The FEA method was employed to simulate the nanoimprinting process using positive and negative molds with aspect ratios of 1:1, 3:1, 5:1, and 7:1. During the filling process, the resist adjacent to boundaries has the maximum displacement. The corners of contact areas between the protruding part of the mold and the resist has the maximum Von Mises stress. For both positive and negative molds, the maximum stress in the mold increases with aspect ratio. However, filling up negative molds is more difficult than positive ones. With the same aspect ratio, the maximum stress in a negative mold is approximately twice as large as that in a positive one.

## 1. Introduction

Nanoimprint lithography (NIL), which overcomes the limitations of conventional optic lithography, is a low-cost and high-throughput technique for manufacturing nanoscale patterns. Fabrication of three-dimensional (3D) high aspect ratio (HAR) nanopatterns has a broad range of applications in many fields, such as superhydrophobic structures [1], optics [2], electronics [3], and biology [4].

Many research groups have fabricated HAR micro- and nanostructures using NIL. Deng et al. employed an anodic aluminum oxide (AAO) template with an ordered deep hole array to fabricate a HAR (>100:1) thiol-ene nanopillar array upon curing under ultraviolet (UV) irradiation [5]. The low-viscosity liquid thiol-ene can overcome the surface tension and becomes a rigid cross-linked polymer after UV curing. Karlsson et al. fabricated HAR nanostructures in polycrystalline diamond using NIL with a feature size of 300 nm and a depth of 2 μm [6]. HAR patterns were fabricated employing a newly developed Si-containing thermal NIL resist by Messerschmidt et al. [7]. Lee et al. used thermal NIL to fabricate a GaN light-emitting diode (LED) with three highly refractive patterned structures: submicron holes, microconvex arrays, and HAR pillars [8]. They found that HAR pillars had the best ability to increase the light extraction efficiency of LEDs. Because Si stamp has a short lifetime, Park et al. fabricated a CoNi mold with HAR structures [9], which was then applied to a 20 nm fluorocarbon antistiction layer. Hirai et al. proposed double-layer resist nanoimprinting to fabricate HAR structures under low-pressure conditions [10]. This process can eliminate the occurrence of fracture defects in the stamp-releasing step.

Completion of the filling process using NIL with a HAR mold is more difficult than with normal-mold NIL. A HAR mold may not be ideally replicated, and defects increase with aspect ratio [11]. Because of this, more theoretical and simulation analysis work needs to be done in lieu of experimentation, since experiments consume expensive stamp and resist. Hirai et al. proposed the Maxwell equation to study polymer filling according to the imprint temperature and pressure [12]. The finite-element method (FEM), which is also referred to as finite-element analysis (FEA), is a numerical technique for finding approximate solutions by subdividing a large problem into a smaller and simpler one. To understand delamination, buckling, and fracture in the nanoimprinting process, Hsueh et al. identified factors that result in mechanical failure by simulating stresses based on the FEA method [13]. Lee et al. [14] obtained a numerical viscoelastic material model for polycarbonate (PC) and simulated the micro-thermal-imprinting process using PC near the glass transition temperature (T_g_) using the FEA method. Zou et al. simulated the de-molding behavior of nanochannels during hot embossing process based on FEM [15]. To understand the behavior of the imprint polymer under low-temperature nanoimprinting, FEM simulations of thin polymer films squeezing into stamp cavities were performed by Sin et al. during NIL with a temperature range T_g_ < T < T_g_ + 40 °C using a two-dimensional viscoelastic model [16]. H. D. Rowland and W. P. King conducted the resist deformation embossing with the temperature around T_g_ −10 < T < T_g_ + 20 °C. They pointed out that cavity size has great influence on the polymer deformation [17]. Xie et al. [18] combined thermal NIL and a high-temperature transferring technique to fabricate a moiré grating. They used FEM to optimize the grating depth by analyzing the stress intensity. FEA simulations were performed by Liu et al. to investigate the nanoimprinting properties of bilayer substrates of an aluminum/polymide [19]. They studied the effects of the bilayer thickness ratio and temperature on the pressure needed to completely fill the stamp cavities. Ding et al. imprinted ultrafiltration (UF) membranes, and FEA simulations were carried out to understand the deformation mechanism of the membrane during nanoimprinting [20]. Sonne et al. employed FEA to simulate the deformation of flexible polytetrafluoroethylene (PTFE) nanoimprinting stamps, which were used to imprint on curved surfaces [21].

In general, there is a lack of studies of modeling nanoimprinting with HAR molds in the literature. In this paper, FEA was applied to analyze the stamp-cavity-filling process and corresponding stress distribution with different aspect-ratio molds to study the inner resist flow mechanism and each part’s stress when nanoprinting with HAR molds.

## 2. Simulation Models

To save time, 2D FEM models were employed. The following simplifications were assumed for the entire simulation: (1) all molds are rigid-body type, and cannot be deformed by applying an outside force; (2) the imprint resist is an incompressible, isotropic, and nonlinear hyperelastic polymer; (3) there is no slide between mold and resist during imprinting; and (4) surface force is to be neglected. As is common, silicon (Si) and poly(methyl methacrylate) (PMMA) were chosen as mold and resist, respectively. The material properties are shown in Table 1. The T_g_ of PMMA is set to be 105 °C, or 378 K. Usually, the imprint temperature is 50–100 °C higher than the polymer’s T_g_. For better resist filling ability and understanding pressure effect, 480 K imprint temperature was employed in all simulations.

**Figure 1:**
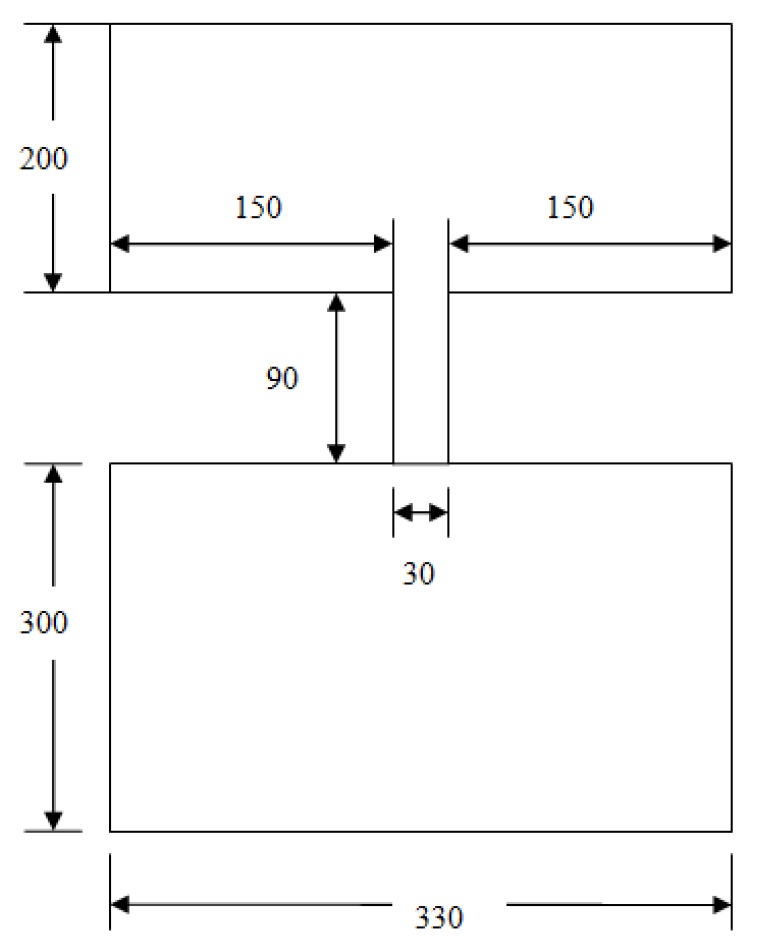
Model of a typical HAR (3:1) mold and resist (units of nm).

Both positive and negative molds with aspect ratios of 1:1, 3:1, 5:1, and 7:1 were set up to simulate the filling process. Von Mises stress is used to evaluate the stress distribution in all kinds of molds. One typical model, with a mold aspect ratio of 3:1, is shown in Figure 1. In this model, the length of the mold protrusion is 30 nm and its height is 90 nm. The lengths of the left- and right-hand sides of the protrusion are both 150 nm. The base height of the mold is 200 nm. The resist length is 330 nm and its height is 300 nm. For other positive molds, only the protrusion height was changed while other parameters remained unchanged; the same conditions were true for negative molds. In all the simulations, the time period is set at 1 s.

The pressure employed in this contribution is at least 20 times higher than other references, such as 50 MPa in [22]. This is because high aspect ratio stamps, compared to traditional stamps, find it harder to fully fill all the cavities, especially the corners, as analyzed in the following section.

## 3. Results and Discussion

### 3.1. Mold-Cavity-Filling Analysis

**Figure 2:**
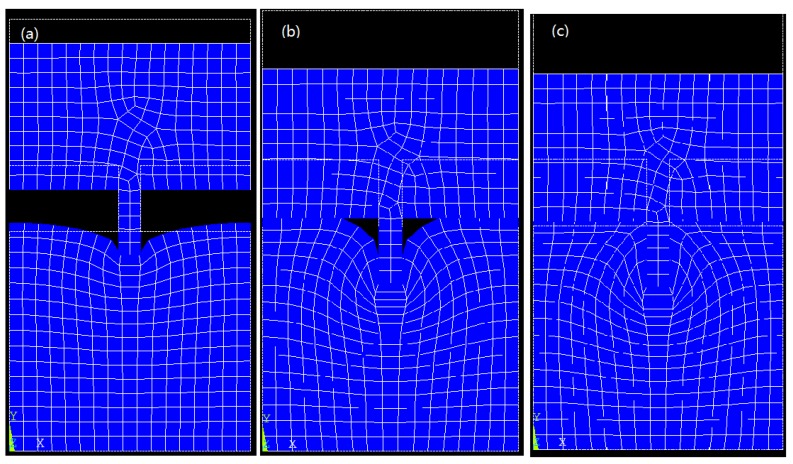
Positive-mold-filling ratio for an aspect ratio of 3:1 increasing with imprinting pressure: (**a**) 1 × 10^8^, (**b**) 5 × 10^8^, and (**c**) 1.4 × 10^9^ Pa.

For all molds with different aspect ratios, the cavities can only be filled to a small degree when a low imprinting force is applied. With the same imprint temperature, 480 K, many imprint simulation tests were conducted, each time with a higher imprint force until all cavities in the mold were fully filled up. Taking a positive mold with an aspect ratio of 3:1 as an example, only a small portion of the cavities were filled when the imprint pressure was 1 × 10^8^ Pa. When the pressure was increased to 5 × 10^8^ Pa, most of the cavities were filled up. Until the pressure reached 1.4 × 10^9^ Pa, the mold pattern was faithfully replicated to the resist, as shown in Figure 2. It was found that after most of the cavities were filled up, it was comparatively harder to fully fill the remaining small unfilled part of the cavities, especially in the corners.

**Figure 3:**
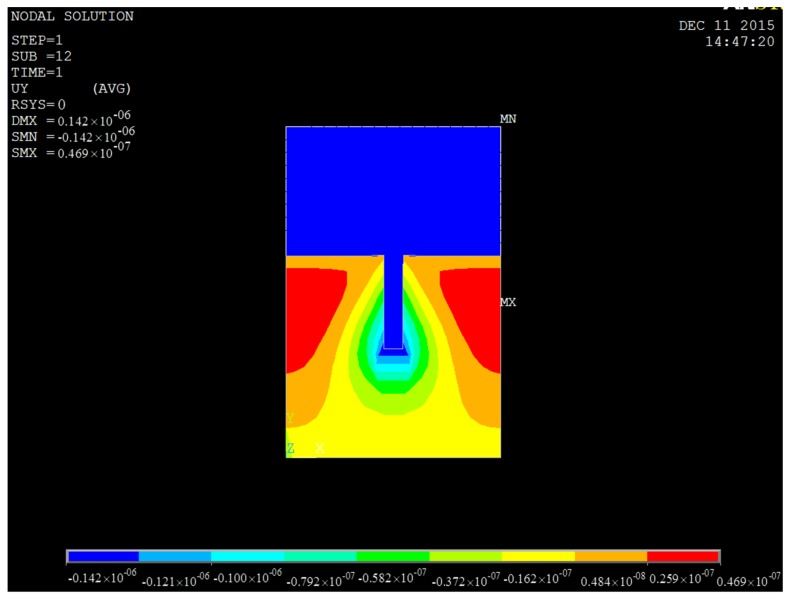
Displacement of a positive mold with an aspect ratio of 5:1, and the corresponding resist.

In addition, the mold and resist displacement were also investigated. Figure 3 shows the displacement of a positive mold with an aspect ratio of 5:1 and the corresponding resist. The color and scale bar denote different displacement values; red and blue indicate maximum and minimum displacement, respectively.

**Figure 4:**
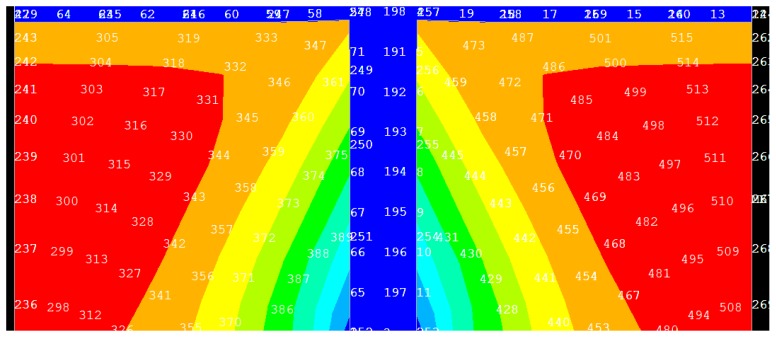
Part of resist displacement (magnified) from Figure 3.

**Figure 5:**
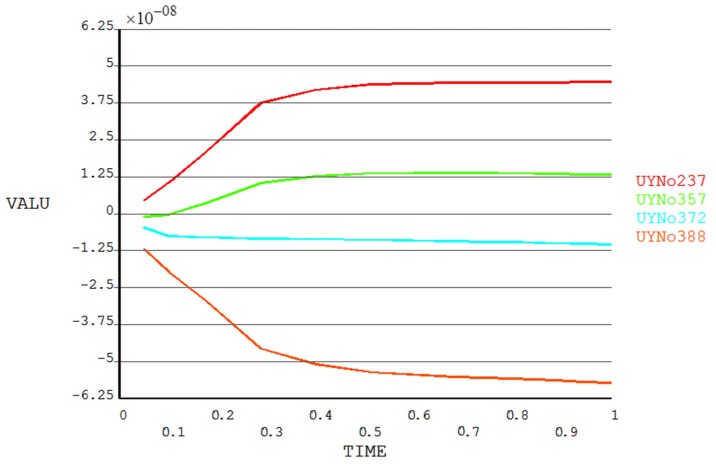
Displacement change with imprinting time of nodes 237, 357, 372, and 388.

We next focused on the resist flow and filling into the cavities of the mold. The magnified resist part of Figure 3 is shown in Figure 4, with node numbers marked. Owing to symmetry, only the left-hand part of the resist was analyzed. Four typical nodes, numbers 237, 357, 372, and 388, were chosen, and Figure 5 plots the displacement change with imprinting time for these four nodes. From the figures, it can be concluded that, during the whole imprinting process, the leftmost part of the resist had the maximum displacement. This part is far from the protrusion part of the mold, or, in other words, the center of the model. For other resists, the closer to the center, the smaller the displacement. During imprinting, the displacement of the left-hand part of the resist increases gradually with imprinting time. Until the resist flow is almost completed, the displacement remains nearly stable. However, for those resist parts close to the center, the initial displacement is obviously larger than in the latter period. When imprinting is nearly finished, the displacement of this part of the resist is also close to stable. The reason for this phenomenon is that the resist needs to travel a long distance in order to fill the cavity in the initial stage. In the latter period, the displacement change is very small since the majority of the cavity has been filled by the resist.

The filling and displacement analyses for molds of other aspect ratios have been omitted because they show the same regularity.

### 3.2. Von Mises Stress Analysis

**Figure 6:**
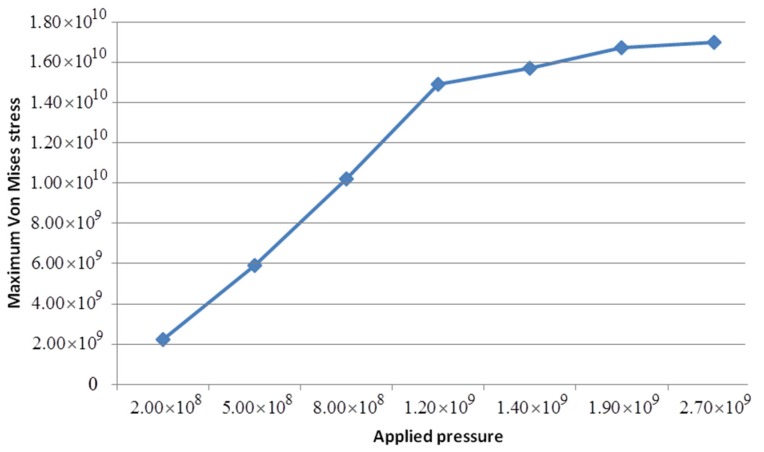
Relationship between the maximum Von Mises stress and imprinting pressure (units of Pa).

For stress analysis, a positive mold with an aspect ratio of 7:1 was taken as representative. Table 2 lists the maximum Von Mises stresses under different imprinting pressures. Figure 6 shows the relationship between the maximum Von Mises stress and imprinting pressure, indicating that the maximum stress increases with applied pressure.

**Figure 7:**
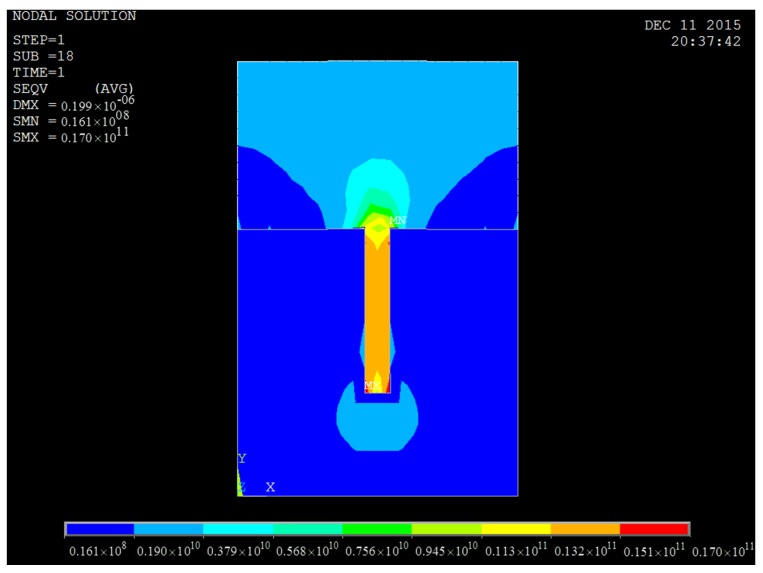
Von Mises stress distribution for a positive mold with an aspect ratio of 7:1, and the corresponding resist.

**Figure 8:**
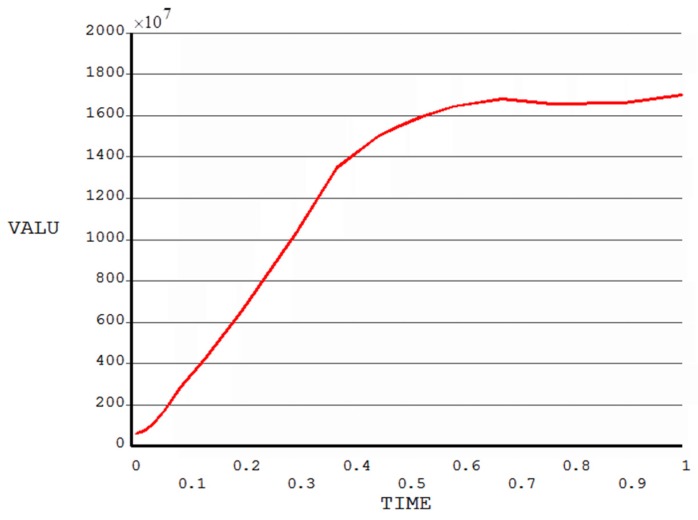
Relationship between Von Mises stress and imprinting time at the node undergoing maximum Von Mises stress.

When the imprinting pressure reached 2.7 × 10^9^ Pa, the filling of the cavities of the positive mold with a 7:1 aspect ratio was just being completed. In this case, the Von Mises stress distributions for both mold and resist are shown in Figure 7, in which it is noticeable that the maximum Von Mises stress always appears at the corners of contact areas between the protrusion part of mold and the resist, regardless of the mold’s aspect ratio. It was also found that the Von Mises stress of both the resist and the stamp increased with the imprint pressure applied. Figure 8 shows the relationship between Von Mises stress and time at the node undergoing maximum Von Mises stress with an imprinting pressure of 2.7 × 10^9^ Pa for a positive mold with an aspect ratio of 7:1. The stress increases with time until the maximum value is reached at 1.70 × 10^10^ Pa. At the first half period, the stress increased sharply and in the second half period the stress became stable gradually. This is because stress accumulates on the corners of the stamp in the first period and has no time to release the stress. When most of the resist has occupied the cavities of the stamp, the newly generated stress and release of existing stress are gradually balanced.

### 3.3. Comparison among Different Molds

**Figure 9:**
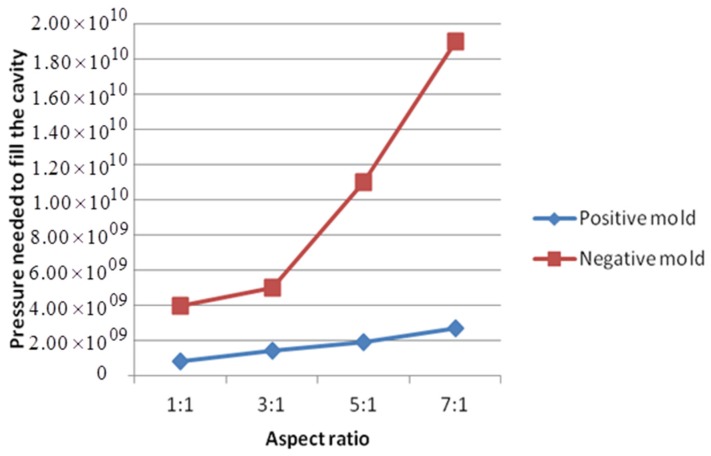
Relationship between pressures needed to fill cavities and different aspect ratios for both positive and negative molds (units of Pa).

The imprinting pressures needed to only just completely fill the cavities of different molds are summarized in Table 3 and Figure 9, which indicate that filling negative molds is more difficult than positive ones. Negative molds need higher pressure to fully transfer patterns. The higher the aspect ratio, the more obvious this phenomenon is.

**Figure 10:**
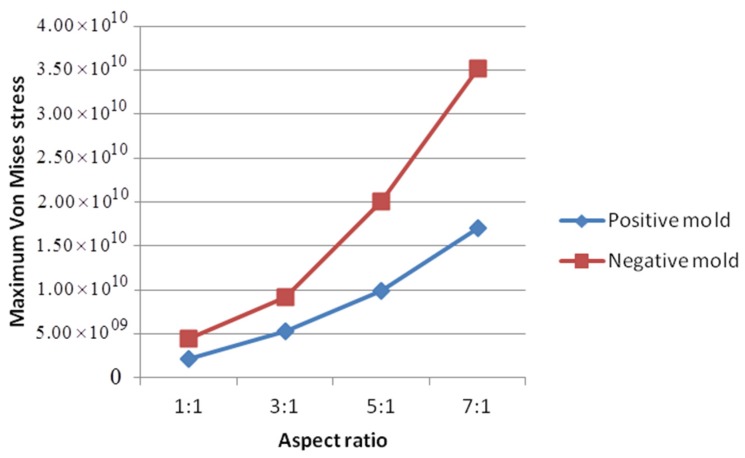
Maximum Von Mises stresses in completely filled different molds (units of Pa).

When the resist fully occupied the mold cavities, the maximum Von Mises stresses for positive and negative molds are summarized in Table 4 and Figure 10. For both positive and negative molds, the maximum stress in the mold increases with aspect ratio. For a negative mold, this increase is more obvious. With the same aspect ratio, the maximum stress in a negative mold is approximately twice as large as in a positive one. Therefore, designing a positive HAR mold will help reduce the stress intensity on the mold compared to a negative one.

## 4. Conclusions

High aspect ratio (HAR) nanopatterns have many applications in optics, electronics, biology, and other fields. Since nanoimprint lithography (NIL) is a low-cost, high-throughput, and high-resolution pattern-replication technique, it is practical to fabricate HAR nanostructures. However, completing the filling process using NIL with a HAR mold is more difficult. Therefore, employing finite-element analysis (FEA) to simulate the NIL process can help to eliminate expensive experimental costs and reveal detailed information on the resist-flow mechanism and the stress on different parts of a mold. FEA was applied to analyze the mold-cavity-filling process and corresponding stress distribution of positive and negative molds with aspect ratios of 1:1, 3:1, 5:1, and 7:1. It was found to be comparatively more difficult to completely fill the corners of small cavities. The boundary parts of the resist have a larger displacement than the center parts. Negative molds need higher pressure to fully transfer patterns compared to positive ones, especially under HAR conditions. The maximum Von Mises stress always appeared at the corners of contact areas between the protrusion part of a mold and the resist. The maximum stress in the mold increased with aspect ratio for both positive and negative molds. However, the maximum stress in a negative mold was approximately twice as large as a positive one with the same aspect ratio. Therefore, it is a good idea to design a positive HAR mold with round corners if conditions allow.

## Figures and Tables

**Table 1:** Material properties for a Si mold and PMMA resist.

Materials	Elastic Modulus (GPa)	Poisson’s Ratio	Density (kg/m^3^)	Thermal Conductivity Coefficient	Specific Heat Capacity (J/kg)
Si	190	0.300	2330	149.00	700
PMMA	3	0.499	1190	0.21	1465

**Table 2:** Maximum Von Mises stresses under different imprinting pressures (units of Pa).

Applied pressure	2.0 × 10^8^	5.0 × 10^8^	8.0 × 10^8^	1.2 × 10^9^	1.4 × 10^9^	1.9 × 10^9^	2.7 × 10^9^
Maximum stress	2.24 × 10^9^	5.92 × 10^9^	1.02 × 10^10^	1.49 × 10^10^	1.57 × 10^10^	1.67 × 10^10^	1.70 × 10^10^

**Table 3:** Pressures needed to just completely fill up the cavities of different molds (units of Pa).

Aspect Ratio	1:1	3:1	5:1	7:1
Positive mold	8 × 10^8^	1.4 × 10^9^	1.9 × 10^9^	2.7 × 10^9^
Negative mold	4 × 10^9^	5.0 × 10^9^	1.1 × 10^10^	1.9 × 10^10^

**Table 4:** Comparison of maximum Von Mises stresses for positive and negative molds (units of Pa).

Aspect Ratio	1:1	3:1	5:1	7:1
Positive mold	2.10 × 10^9^	5.25 × 10^9^	9.87 × 10^9^	1.70 × 10^10^
Negative mold	4.44 × 10^9^	9.21 × 10^9^	2.00 × 10^10^	3.51 × 10^10^
